# In This Issue

**Published:** 1999

## UNDERSTANDING STRESS: CHARACTERISTICS AND CAVEATS

Exposure to stressful situations—from unexpected calamities to routine daily annoyances—is a universal human experience. Stress can cause physiological and psychiatric disorders, and has been postulated to promote relapse among alcoholics, even after months or years of abstinence. In response to stress, the body mobilizes an integrated complex of psychological and biochemical reactions apparently designed to restore the body’s equilibrium. Under certain circumstances, however, the body’s response can actually exacerbate the adverse effects of stress. Drs. Hymie Anisman and Zul Merali describe research on the stress response, focusing on processes potentially relevant to the development of alcohol dependence and abuse. The authors provide evidence to show that the outcome of the stress response depends on characteristics of the stressor (e.g., controllability, predictability, and timing of exposure) and of the stressed organism, whether human or animal (e.g., age, gender, genetic factors, and previous stressor exposure). A better understanding of the multiplicity of possible interactions among these factors could support efforts to prevent both the initiation and re-initiation of problem drinking. (pp. 241–249)

## DOES DRINKING REDUCE STRESS?

Drinking to “unwind” is a common activity for many people. Indeed, many researchers believe that alcohol’s anticipated stress-relieving effect is what prompts most people to consume alcohol. Yet findings on exactly how alcohol affects stress have been inconsistent. In this article, Dr. Michael A. Sayette reviews the mechanisms responsible for regulating alcohol’s stress-response dampening (SRD) effect. A number of factors appear to mediate the SRD effect of alcohol; those factors vary from person to person and by situation. For example, individual differences that might influence the SRD effect include a family history of alcoholism, certain personality traits, and gender. Situational factors also influence the drinker’s response to alcohol. That is, the same person experiences alcohol’s effects differently when drinking at a party with friends versus drinking alone at a bar after a stressful day at work. The author cites two models to further describe those situational factors and the role that they may play in a person’s use of alcohol to cope with stress. (pp. 250–255)

## THE ROLE OF UNCONTROLLABLE TRAUMA IN THE DEVELOPMENT OF PTSD AND ALCOHOL ADDICTION

Why do some people develop problems with alcohol after experiencing a traumatic event? Dr. Joseph Volpicelli, Ms. Geetha Balaraman, Ms. Julie Hahn, Ms. Heather Wallace, and Dr. Donald Bux present a model to help explain how a traumatic event may influence alcohol consumption. According to this model, victims of trauma later experience adverse consequences—such as posttraumatic stress disorder (PTSD) and alcohol dependence—depending on the degree to which they were able to take control of the event. People who have experienced uncontrollable trauma may turn to alcohol in an attempt to relieve symptoms of PTSD. The irony is that while alcohol use may temporarily relieve the symptoms of stress, alcohol withdrawal intensifies them. The patient then is caught in a cycle in which he or she must continue to drink to stave off the recurrence of increasingly worse symptoms. (pp. 256–262)

## THE ROLE OF STRESS IN ALCOHOL USE, ALCOHOLISM TREATMENT, AND RELAPSE

The notion that exposure to stressful situations in everyday life can cause susceptible people to initiate or relapse to drinking seems logical. Yet in the clinical arena, the exact relationship between stress and alcohol use has been difficult to characterize. Drs. Kathleen T. Brady and Susan C. Sonne explore the relationship among stress and alcohol use, alcoholism treatment, and relapse. The authors also attempt to identify and explain the role that key brain chemicals (or mediators) may play in the alcohol-stress relationship. These mediators may include serotonin, dopamine, and opiate peptide systems, as well as the stress-response system itself—the hypothalamic-pituitary-adrenal (HPA) axis. As stated by the authors, further exploration of these mediators should lead to important pharmacological developments in the prevention and treatment of alcohol and other drug abuse. In addition, treatment approaches that emphasize stress management strategies also are likely to prove more beneficial, especially for patients who have previously relapsed to drinking in response to stressful situations. (pp. 263–271)

## ALCOHOL, AGING, AND THE STRESS RESPONSE

The body responds to a stressful situation by launching a series of neurochemical events that ultimately result in the production of a key class of hormones (i.e., glucocorticoids) to counter the stressful situation. Too much of a good thing can be detrimental, however, as excess glucocorticoid production produces a host of harmful effects on the body. Drs. Robert L. Spencer and Kent E. Hutchison investigate one of the causes of excess glucocorticoid secretion—alcohol intoxication. Ironically elevated levels of these stress hormones may actually contribute to alcohol’s pleasurable effects. Chronic drinking, as well as chronic glucocorticoid exposure, however, can result in premature and/or exaggerated aging. The aging process, in turn, may affect a person’s sensitivity to alcohol and stress hormone production. Thus a three-way interaction exists among alcohol consumption, stress hormone production, and the aging process. This article examines this three-way relationship. By highlighting the overlap among these relationships, the authors hope to spur further research on this important and complex topic. (pp. 272–283)

## WORK STRESS AND ALCOHOL USE

Stress in the workplace is a common factor. Can a stressful job lead to problems with alcohol? Dr. Michael R. Frone reviews four research models that have been developed to explain the effects of stress on employee drinking: the simple cause-effect model, the mediation model, the moderation model, and the moderated mediation model. Research clearly supports a relationship between work-related stressors (such as job demands, job control, and job complexity) and elevated alcohol consumption and problem drinking. Future research on this topic should include a focus on work stressors and alcohol use among adolescents and young adults; this group is just entering the workforce and has been found to be most likely to engage in heavy drinking. Longitudinal studies also are needed to better explain the long-term relationship between work stress and alcohol use. (pp. 284–291)

## CAN YOUR CHILDREN DRIVE YOU TO DRINK?

Many parents acknowledge that their children can be a source of stress. Not surprising, parents of children with behavior problems—particularly attention deficit hyperactivity disorder (ADHD)—report very high levels of daily child-rearing stress. Several investigations have addressed parenting stress caused by disruptive children, yet only a handful of studies have examined how parents cope with this stress and whether alcohol use is higher in those parents. Drs. William E. Pelham, Jr., and Alan R. Lang review what is known about childhood behavior problems and subsequent parental drinking behavior. Given that parenting hassles may result in increased alcohol consumption even in “normal” families, the authors conclude that the stress associated with parenting and its influence on alcohol consumption warrants further research as part of the larger study of stress and alcohol use. (pp. 292–298)

## A LONGITUDINAL STUDY OF STRESS, ALCOHOL, AND BLOOD PRESSURE IN COMMUNITY-BASED SAMPLES OF BLACKS AND NON-BLACKS

Research supports the theory that people drink to relieve stress. Yet because both stress and alcohol use are associated with increased blood pressure, drinking to cope with stress may not only lead to alcohol problems but may increase the risk for high blood pressure and its associated problems. Drs. Marcia Russell, M. Lynne Cooper, Michael R. Frone, and Robert S. Peirce examine the relationships among stress, alcohol use, and blood pressure using findings from a series of surveys. The researchers identify factors that help reduce the impact of stressors on alcohol use as well as factors that may increase the impact. To determine if these factors differ by race or by gender, the authors also examine findings for blacks compared with non-blacks and for men compared with women. (pp. 299–306)

